# Application and teaching of computer molecular simulation embedded technology and artificial intelligence in drug research and development

**DOI:** 10.1515/biol-2022-0675

**Published:** 2023-08-11

**Authors:** Xiaoling Chen, Junmin Zhang, Quanyi Zhao, Li Ding, Zhengrong Wu, Zhong Jia, Dian He

**Affiliations:** Materia Medica Development Group, Institute of Medicinal Chemistry, Lanzhou University School of Pharmacy, Lanzhou 730000, China; The NO. 2 People’s Hospital of Lanzhou, Lanzhou 730000, China

**Keywords:** artificial intelligence, drug development, computer technology, molecular simulation

## Abstract

With the continuous development of the pharmaceutical industry, people have always paid attention to the safety and effectiveness of drugs, including innovative drugs and generic drugs. For pharmaceutical companies as manufacturers, drug development is a very lengthy process that requires high costs, millions of man-hours, thousands of trials, and the mobilization of hundreds of researchers. Therefore, efforts need to be made to develop drugs with high safety and effectiveness. Drug research and development plays an important role today. Based on this, this article applied computer molecular simulation embedded technology and artificial intelligence technology to drug research and development. First, the problems faced in the research and development of anti-inflammatory disease-dependent tumor drugs were introduced, and then the applications of computer molecular simulation embedded technology and artificial intelligence technology in drug research and development were analyzed. Subsequently, the application of artificial intelligence in drug research and development teaching was analyzed, and a teaching system based on computer molecular simulation embedded technology and artificial intelligence was designed. Finally, the application effects of computer molecular simulation embedded technology and artificial intelligence technology were analyzed, and a feasible conclusion was drawn. The use of computer molecular simulation embedded technology and artificial intelligence technology can greatly improve the efficiency of drug research and development, and the research and development safety of imatinib mesylate has been improved by 7%. On the other hand, it can improve students’ learning interest and stimulate their learning interest, and students’ drug research and development capabilities have been improved. Drug research and development for inflammatory-dependent tumors has good application prospects.

## 1 Introduction

There are many difficulties in drug research and development, and few people in the field of inflammatory-dependent tumor research have conducted a detailed analysis of drugs, elaborating on the design, synthesis, and mechanism of action of innovative drugs for inflammatory-dependent tumor, which has led to strong obstacles in the development of innovative drugs for inflammatory-dependent tumor. To remedy this defect and let more people understand drug research and development work, it is necessary to use new technologies to analyze drug research and development.

Drug research and development has received widespread attention from the public, and many scholars have made achievements in this field. Mak Kit-Kay discussed the main reasons for the loss rate of new drug approval, the possible ways in which artificial intelligence could improve the efficiency of the drug development process, and the collaboration between pharmaceutical industry giants and artificial intelligence drug research and development companies [1]. Dugger et al. first examined and emphasized the successes and limitations of the early stages of genomics in drug research and development. He then reviewed the current major efforts in precision medicine and discussed the potential wider uses of mechanically guided therapy in the future [2]. Chaikuad reviewed the latest developments in this rapidly growing area of kinase drug development and highlighted the unique opportunities and challenges of this strategy [3]. Devine discussed some key drivers and scientific developments that were expanding the application of biocatalysis in the pharmaceutical industry, and highlighted potential future developments that might continue to increase the impact of biocatalysis in drug development [4]. Kraus described the salient features of biomarker discovery, analytical validation, clinical identification, and utilization to understand the development process of biomarkers. He also conveyed his understanding of its potential advantages and limitations through this understanding [5]. Weissig analyzed the etiology and pathogenesis of mitochondrial diseases over the past two decades and applied them to the design and development of new experimental drugs for treating mitochondrial diseases [6]. Cochran et al. reviewed the current status of the biology and clinical development of bromine domain and extracellular inhibitors, and discussed the next wave of bromine domain inhibitors with clinical potential in oncology and nononcology indications [7]. The application of drug research and development is rarely studied in inflammatory-dependent tumors.

Artificial intelligence technology has excellent application effects in the field of drug research and development. Fleming explored how artificial intelligence could change drug development [8]. Schneider introduced the views of different international expert groups on the “significant challenges” of artificial intelligence small molecule drug research and development and the methods to solve these problems [9]. Chan believed that the combination of artificial intelligence and new experimental technologies was expected to make the search for new drugs faster, cheaper, and more effective. He discussed the emerging applications of artificial intelligence in improving the drug development process [10]. Yang comprehensively described these machine-learning technologies and their applications in pharmaceutical chemistry [11]. He reviewed some key practical issues surrounding the implementation of artificial intelligence in existing clinical workflows, including data sharing and privacy, transparency of algorithms, data standardization and interoperability across multiple platforms, as well as attention to patient safety [12]. Stephenson understood the current situation of machine-learning technology in the academic and industrial environment of drug research and development, and discussed its potential future applications and several interesting models of machine-learning technology in the field of drug research and development [13]. Harrer explained how to use the latest advances in artificial intelligence to reshape the key steps of clinical trial design to improve the trial success rate [14]. Artificial intelligence technology has rarely been studied in combination with computer molecular simulation embedded technology.

To improve the effectiveness of innovative drug research and development for inflammatory-dependent tumors, this article applied computer molecular simulation embedded technology and artificial intelligence technology to drug research and development. Four typical antitumor drugs were selected to analyze their application effects, and five students who studied the design, synthesis, and mechanism of action of innovative inflammatory-dependent tumor drugs were selected to analyze their teaching effects. Finally, it was concluded that the use of computer molecular simulation embedded technology and artificial intelligence technology can greatly improve the application and teaching effectiveness of drug research. Compared with other people’s experiments, this article combined computer molecular simulation embedded technology and artificial intelligence technology to apply.

## 2 Problems faced by the research and development of anti-inflammatory-dependent tumor drugs

**Figure 1:**
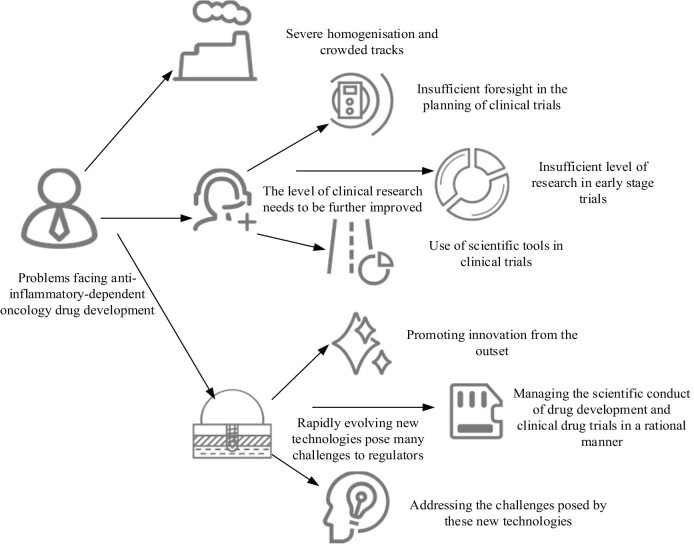
Problems faced in the development of anti-inflammatory-dependent oncology drugs.

The problems faced by the research and development of anti-inflammatory-dependent tumor drugs include competition among peers, lack of clinical research, and lax supervision, as shown in Figure 1.

### 2.1 Serious homogenization and circuit congestion

Currently, the research and development of anti-inflammatory tumor drugs is facing serious homogenization and the threat of race track congestion, and there is a relatively detailed division of anti-inflammatory tumor drugs in different race tracks. Moderate competition between these pathways is beneficial to the development of drug research and development, not only stimulating research and development and promoting industrial development but also promoting the reduction of drug prices and thus benefiting patients. However, issues such as pseudo-innovation, crowdsourcing, and low-level repetitive construction still require high attention from relevant sectors.

### 2.2 Level of clinical research needs to be further improved

After the standardized development of drug review and approval reform, clinical trial capabilities have made great progress. However, it should be clearly recognized that clinical trials are transitioning from generic drugs to innovative drugs, and there is still a significant gap between the level of clinical trials of new inflammatory-dependent tumor drugs and the level of clinical trials in developed countries. In key clinical stages, the selection of control drugs is unreasonable, and there is a lack of understanding of real-world research. The selection and evaluation of clinical endpoints is not scientific, and the participation and attendance rate of international multicenter clinical trials are not high. The application of modern research models/tools is relatively late, and the selection of research populations lacks scientific nature.

Clinical trials are one of the most critical steps in the development of innovative drugs, as well as an important step in transforming the biological determinants of cancer into treatment options, providing the most convincing and reliable evidence for the safety and effectiveness of drugs. If clinical trials cannot be further improved to meet international standards and comply with the current trend of slowing tumor growth, it may not only affect the smooth and efficient development of new drugs, especially in the field of anticancer drug development, but will also challenge many scientific and ethical issues for a long time.

### 2.3 Rapid development of emerging technologies has brought many challenges to regulatory authorities

Innovation is the core of research and development. In recent years, the emergence of cell and gene therapy technologies, new drug delivery technologies, and other innovative technologies has changed the concept and regulatory evaluation of new inflammatory-dependent tumor drugs, triggering a new round of research and development boom, and the global pharmaceutical landscape has undergone significant changes. Unlike chemical drugs and ordinary biological agents, they differ significantly from other existing drugs in terms of product manufacturing, process complexity, *in vivo* biological characteristics and safety risks, as well as personalized use, which poses many challenges and significant regulatory uncertainty. Due to this disruptive application technology being at the forefront of life science research, academia, industry, and regulatory agencies have reached a stage where they do not fully understand the laws of basic theoretical research, transformation research, and clinical application. How to promote innovation from the beginning, how to manage the scientific conduct of drug development and clinical drug trials in a reasonable manner, taking into account the needs of patients, and how to address the challenges posed by these new technologies are requirements of the existing regulatory framework and the technical evaluation system. The key challenge for regulators in this field is to scientifically evaluate and regulate the products of these new technologies.

## 3 Application of computer molecular simulation embedded technology and artificial intelligence technology in drug research and development

### 3.1 Application of computer molecular simulation embedded technology

The gradual development of computer molecular simulation embedded technology and artificial intelligence technology has brought drug development into the stage of rational drug design. Based on the results of biochemistry, molecular biology, genetics, computer science, and computational chemistry, potential targets for drug design such as enzymes, receptors, and ion channels have been identified, and the chemical structures of ligands or natural substrates belonging to other categories have been taken into account to rationally develop drugs.

The technology of using computer graphics for molecular modeling is called computer molecular simulation embedded technology. Computer molecular simulation embedded technology uses computers to construct, represent, and analyze molecular models to visualize molecular structures, and simulates the three-dimensional conformation of molecules to visualize the interactions between small drug molecules and biomacromolecules. By identifying potential active sites where small drug molecules bind to receptor macromolecules, the structure of small molecules is modified to find better solutions for improving drug design, making drug research and development intuitive and insightful [15].

Computer molecular simulation embedded technology can provide information about molecular docking: three-dimensional structure of molecules, physical and chemical properties of molecules, structural comparisons between molecules, conformational changes of molecules, flexibility and kinetic properties, and the shape of drug target complexes. Therefore, by using molecular modeling, we can observe and analyze three-dimensional molecular models, and study the adaptability and interaction between drugs and targets, which is an important tool for studying the three-dimensional structure of molecules and using molecular docking to study targets and precursors.

The computer molecular simulation embedded technology in virtual drug screening can effectively improve the current technical status of drug screening. The so-called virtual filtering is a computer-based filtering technology. In this technology, preliminary computer screening greatly reduces the number of drug molecules that need to be screened in reality, enhancing the search for lead compounds. Virtual screening can be used to predict the potential activity of drug molecules, detect potential compounds, and develop collections of compounds with appropriate characteristics. It represents the virtualization of experimental models and has become a new method and technology for innovative drug research and development [16].

Molecular modeling software is used to simulate interactions between biomacromolecules and their precursors, and to study “descriptors” such as electrostatic field, hydrophobic field, hydrogen bond distribution, general conformation, and chemical structural characteristics of drug binding sites. Descriptors are used to calculate and analyze the affinity and binding properties between two substances to optimize and modify conductive compounds, so as to improve drug–receptor interactions and drug bioavailability, and ultimately make them candidates for drugs.

### 3.2 Application of artificial intelligence technology in drug research and development

As a branch of computer science, artificial intelligence is mainly used to simulate and expand the functions of the human brain. It can be understood as a computer system with human knowledge and behavioral awareness. It can use its own learning and reasoning abilities to solve complex problems to gain experience in solving them and form memories, thereby better understanding human natural language. Artificial intelligence can become a solution for people to take specific actions when encountering events that trigger reactions or when considering complex issues and making decisions. The process of human problem-solving is formalized using graphical methods. Through computer algorithms and programming, computers learn to use this method to solve more complex problems. This set of hardware and software systems used to solve problems is called artificial intelligence. Artificial intelligence is not a finished product, but a continuously evolving technology aimed at using machines to help humans solve problems.

Artificial intelligence technology is widely used in systems biology to predict disease targets, analyze large amounts of data, and establish process models. Because target discovery is a branch of molecular biology research, artificial intelligence technology is not the core of finding new targets, but its important role in target discovery is undeniable.

The application of artificial intelligence models in drug research and development faces many challenges in data preprocessing, model selection, and result evaluation. Current artificial intelligence models, especially deep learning models, often require a large number of labeled samples for training, and the requirements for labeled samples are very high. The obtaining of labeled samples in biomedical and drug research and development requires professional knowledge and experimental validation, which is costly. One of the earliest applications of deep learning in biomedicine is to read images from pathological sections. This is because hierarchical deep learning models are very suitable for learning low-level features of samples such as images. By using hierarchical e-learning, deep learning technology can automatically learn high-level image features, thereby avoiding the trouble of manual feature suggestions to a certain extent.

There is also the problem of labeling samples in medical research, but it is very different from pathological images. Data collected in medical research range from high-throughput histological data to various phenotypic and textual data. The integration and analysis of multiple heterogeneous high-dimensional data sources can compensate for problems at the small sample level of a single data source. The technological trend of artificial intelligence is shifting from traditional large sample learning to small sample learning and feedback learning.

There are different methods and technologies at different stages of drug research and development, and each stage has its own advantages and disadvantages. Artificial intelligence technology can be applied to all stages of drug development, including target screening, small molecule screening, design stage, synthesis, and prevalidation. Artificial intelligence technology is gradually getting rid of the traditional targeted research and development model. In the face of massive and heterogeneous data from multiple sources, artificial intelligence technology is increasingly data oriented.

## 4 Application of artificial intelligence in drug research and development teaching

Artificial intelligence is essentially the science and technology that provides knowledge and enhances computer learning capabilities, and the results of which can then be used for medical training. Technologies related to artificial intelligence include decision support systems, knowledge representation, machine learning, artificial neural networks, data mining, expert systems, and many other fields. Among them, expert systems, machine learning, and intelligent decision support systems are more commonly used in medical education.

**Figure 2:**
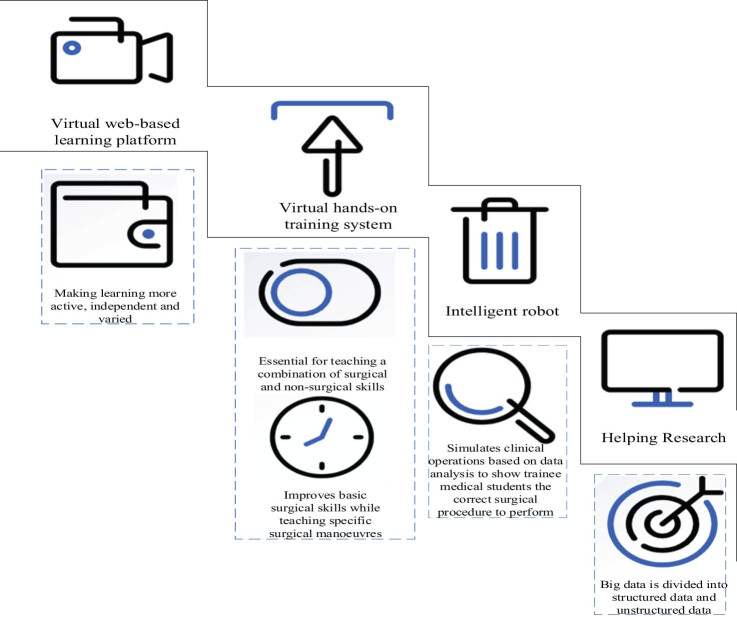
Application of artificial intelligence in teaching drug discovery and development.

In the past decade, many complex educational problems have been solved or partially solved by artificial intelligence, including language processing, reasoning, planning, and cognitive modeling. Artificial intelligence enables students to participate in learning in a digital and dynamic manner, which is often impossible in outdated textbooks or fixed classroom environments. Through this collaborative learning approach, each student has the potential to promote the development of others and accelerate the exploration of new knowledge and the development of innovative technologies. Specific applications are summarized in Figure 2.

### 4.1 Virtual online learning platform

Medical training includes theoretical teaching and clinical practice, so medical students studying inflammatory-dependent tumor drugs need to learn correct clinical techniques while cultivating clinical thinking. Artificial intelligence and big data platforms are used to establish scientific models and methods to evaluate clinical reasoning abilities, which are crucial in medical teaching and practice, and can also be understood as virtual practice. Learning tasks are usually set according to the learning objectives and individualized learning plans of medical institutions and practitioners. Students can interview, examine, assist, diagnose, and treat virtual patients. Then, through interviewing patients, relevant medical reports are obtained, and the condition and plan treatment are determined based on the medical reports. Virtual practice makes learning more active, independent, and diverse.

The emergence of online virtual learning systems not only can rapidly cultivate the ability of medical students in school to solve clinical problems but also can save learning costs, improve learning quality and efficiency, and achieve the sharing of high-quality learning resources. In addition to rapidly cultivating the clinical problem-solving abilities of medical students studying inflammatory-dependent tumor drugs, the virtual online learning system also helps to save teaching costs, improve teaching quality and efficiency, and achieve the sharing of high-quality learning resources.

### 4.2 Virtual training system

The cultivation of medical students studying inflammatory-dependent tumor drugs is inseparable from their practical training, and a good diagnostic foundation is crucial for their future clinical work. The emergence of virtual learning systems can solve the tedious and inactive teaching problems of medical students studying inflammatory-dependent tumor drugs and improve the quality of clinical skill training. Modern, minimally invasive, personalized surgery is attracting great interest. Students can also use this platform to conduct virtual anatomical exercises, simulate different surgical techniques, and create surgical plans. The use of virtual reality technology is crucial for teaching combining surgical and nonsurgical skills, and improving basic surgical skills while teaching specific surgical actions. Medical students studying inflammatory-dependent tumor drugs are able to practice their surgical skills and experience surgical stress and real situations during virtual surgery, which improves the effectiveness of learning.

### 4.3 Intelligent robot

The application of robotics in the medical field is rapidly developing into a new industry, and it is increasingly common to assist surgeons in surgery. The use of robots and virtual reality scenes can also help students conduct surgical training, allowing them to obtain practical training without endangering patients. Robots with artificial intelligence are accurate, stable, and efficient, and can simulate clinical operations based on data analysis to demonstrate correct surgical operations to medical interns.

### 4.4 Supporting scientific research

Scientific research requires artificial intelligence technology to extract large amounts of data. Big data are divided into structured data and unstructured data. Structured data, or inline data, are stored in a database and can be logically represented using a two-dimensional table structure. Unstructured data, including all formats of office files, text, images, various reports, images, and audio/video information, are not simple. The analysis of these data is not simple and requires artificial intelligence technology.

## 5 Teaching system based on computer molecular simulation embedded technology and artificial intelligence

**Figure 3:**
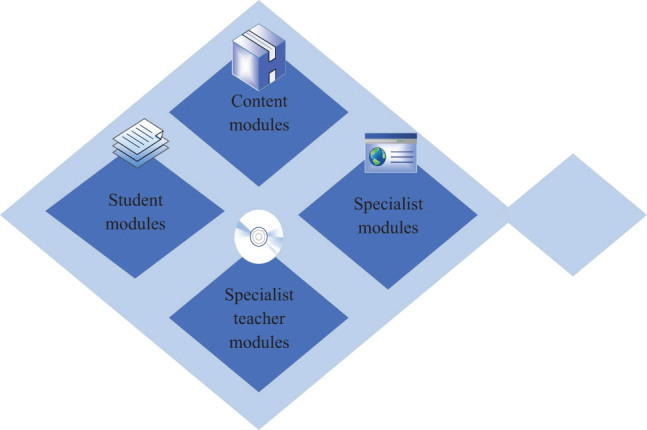
Framework of the teaching system.

Teaching systems based on computer molecular simulation embedded technology and artificial intelligence are divided into content modules, student modules, professional teacher modules, and expert modules, as shown in Figure 3.

### 5.1 Content module

The knowledge in the content module is selected from the database by an expert system, mainly including students’ learning content and teachers’ teaching strategies, guiding the entire teaching process. The learning content includes knowledge content, exercises, and exam content for various disciplines. The content of teaching strategies includes teaching strategies for different teaching periods and is combined with the content of the learner module to develop personalized learning plans for students.

### 5.2 Student module

The student module is the way students use the system. The teaching system is based on molecular computer simulation technology and artificial intelligence, and can record and evaluate students’ learning. Its main function is to collect, record, and analyze information about students’ use of student modules; identify their current strengths and weaknesses in learning; and explain and adjust teaching based on students’ needs. Students’ learning models are established and updated to provide a basis for further teaching. Student learning is recorded, and students are evaluated as a whole.

### 5.3 Professional teacher module

The professional teacher module is mainly used by teachers in the teaching process to guide their work. The knowledge in this module mainly involves teaching strategies. Its main function is to select the optimal teaching strategy based on actual teaching needs and teaching content based on the teaching strategy, read out module information to students, and view students’ learning models. According to the model, it is judged which aspects of knowledge the students lack, so as to modify the teaching strategy and improve the teaching method.

### 5.4 Expert module

The expert module is the core of a learning system based on embedded molecular simulation and artificial intelligence technology, with professional knowledge in education and the ability to answer teaching questions, which can promote learning. Its main functions include the following aspects. First, it reads the student learning information in the student module and selects the most suitable teaching method for each student through intelligent data analysis and reasoning. It can compare and analyze students’ interests and learning habits, and infer their knowledge needs and common mistakes. The learning level of students can be scientifically evaluated, and targeted learning content and teaching methods can be designed. The reasons for students’ errors are comprehensively analyzed and corresponding improvement suggestions are proposed. By utilizing the information from the knowledge base, learning content is formulated according to teaching needs, and the professional and systematic knowledge module content is provided for students to learn and use.

Learning systems based on artificial intelligence differ greatly in knowledge content, teaching strategies, and methods. In learning systems based on computer embedded molecular simulation technology and artificial intelligence, expert modules are constructed to focus on learners’ differences and appropriately evaluate learners’ performance differences in the learning system, thereby enabling learners to learn more effectively. In a learning system based on computer molecular simulation technology and artificial intelligence, the role of a teacher has shifted from being a knowledge imparter and mentor to being a teacher guiding students’ learning. Its main role has shifted from preparing and delivering lessons in the classroom to organizing the learning process, guiding learning habits, and providing the necessary learning environment.

## 6 Application effects of computer molecular simulation embedded technology and artificial intelligence technology

### 6.1 Application effects of drug research and development

In this article, four typical inflammatory-dependent tumor drugs were selected to analyze the effectiveness of drug research and development from the perspective of efficiency and safety. The basic information of the selected drugs is presented in Table 1.

**Table 1:** Application effects of drug development

Selected drugs	Effectiveness
Cyclophosphamide	Influence on DNA structure
Methotrexate	Affects nucleic acid biosynthesis
Docetaxel	Antimitotic
Imatinib mesylate	Based on tumor cell signaling

#### 6.1.1 Efficiency of drug research and development

**Figure 4:**
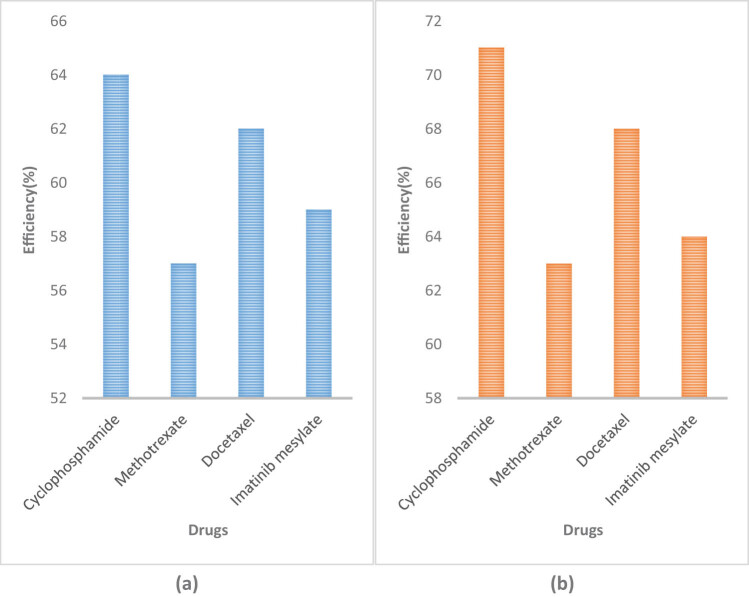
Efficiency of drug development using different methods. (a) Efficiency of drug research using traditional methods. (b) Efficiency of drug research using computerized molecular simulation embedded technology and artificial intelligence.

The efficiency of drug research and development plays a crucial role in the analysis and utilization of drugs. Only by promptly developing drugs that are suitable for patients can they promote their recovery and achieve good therapeutic effects. Based on this, this article used traditional drug research and development methods and used computer molecular simulation embedded technology and artificial intelligence for comparative analysis of drug research methods. The results are presented in Figure 4.

Figure 4a represents the efficiency of drug research and development using traditional methods, and Figure 4b represents the efficiency of drug research using computer molecular simulation embedded technology and artificial intelligence. Using traditional methods, the efficiency of cyclophosphamide research and development was 64%, that of methotrexate research and development was 57%, that of docetaxel research and development was 62%, and that of imatinib mesylate research and development was 59%. However, after using computer molecular simulation embedded technology and artificial intelligence technology, the efficiency of cyclophosphamide research and development was 71%, that of methotrexate research and development was 63%, that of docetaxel research and development was 68%, and that of imatinib mesylate research and development was 64%, which was significantly improved compared to using traditional methods. It can be seen that the combination of computer molecular simulation embedded technology and artificial intelligence technology can greatly improve the efficiency of drug research and development.

#### 6.1.2 Safety of drug research and development

**Figure 5:**
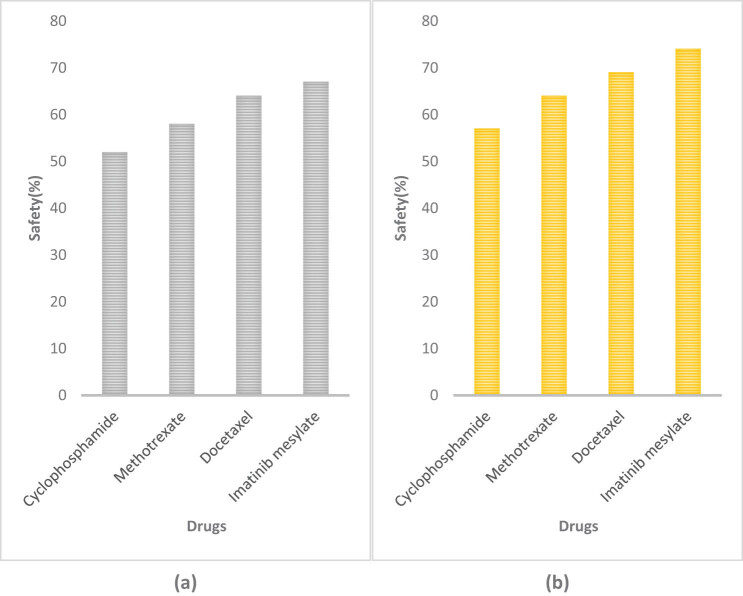
Safety of drug research and development using different methods. (a) Safety of drug research and development using traditional methods. (b) Safety of drug research and development using computer molecular simulation embedded technology and artificial intelligence.

Drug research and development needs to focus on safety issues, as dangerous chemicals and instruments are often used in the process of drug research and development, so it is necessary to study the safety of drug research and development. This article analyzed the safety of drug research and development. The results are shown in Figure 5.

Figure 5a represents the safety of drug research and development using traditional methods, and Figure 5b represents the safety of drug research and development using computer molecular simulation embedded technology and artificial intelligence. The research and development safety of cyclophosphamide has increased from 52% using traditional methods to 57% using computer molecular simulation embedded technology and artificial intelligence technology, increasing by 5%. The research and development safety of methotrexate has increased from 58% using traditional methods to 64% using computer molecular simulation embedded technology and artificial intelligence technology, increasing by 6%. The research and development security of docetaxel has increased from 64% using traditional methods to 69% using computer molecular simulation embedded technology and artificial intelligence technology, increasing by 5%. The research and development safety of imatinib mesylate has increased from 67% using traditional methods to 74% using computer molecular simulation embedded technology and artificial intelligence technology, increasing by 7%. It can be seen that the research and development safety of imatinib mesylate has improved fastest.

### 6.2 Teaching effects of drug research and development

The teaching effect of drug research and development selected medical students from A University who are studying inflammatory-dependent tumors as the subjects of investigation to analyze their drug research and development abilities and drug learning interests. Five students were selected from the survey as representatives for the analysis. This article used a scoring system for the survey of students, with a full score of 100 points.

#### 6.2.1 Student’s interest in drug learning

**Figure 6:**
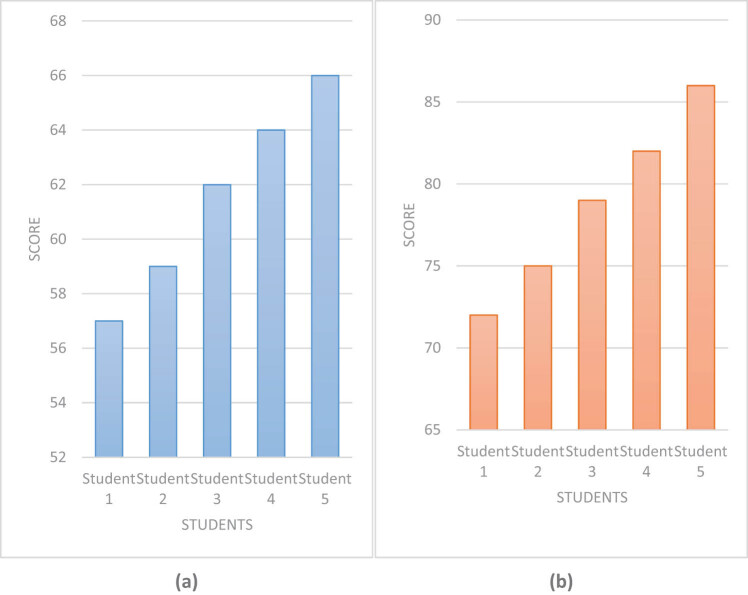
Drug learning interest of students using different methods. (a) Drug learning interest of students using traditional methods. (b) Drug learning interest of students using computerized molecular simulation embedded technology and artificial intelligence.

Interest is the best teacher for learning. Whether students can improve or make progress mainly depends on whether they have interest in learning. This article investigated students’ interest in drug learning, which is presented in Figure 6.

Figure 6a represents the drug learning interests of students using traditional methods, and Figure 6b represents the drug learning interests of students using computer molecular simulation embedded technology and artificial intelligence. When using traditional drug research and development methods, each student’s drug learning interest score did not exceed 70 points. However, after using computer molecular simulation embedded technology and artificial intelligence technology, each student’s drug learning interest score exceeded 70 points. It shows that the use of computer molecular simulation embedded technology and artificial intelligence technology can greatly improve students’ learning interest and stimulate their learning interest.

#### 6.2.2 Student’s drug research and development capabilities

**Figure 7:**
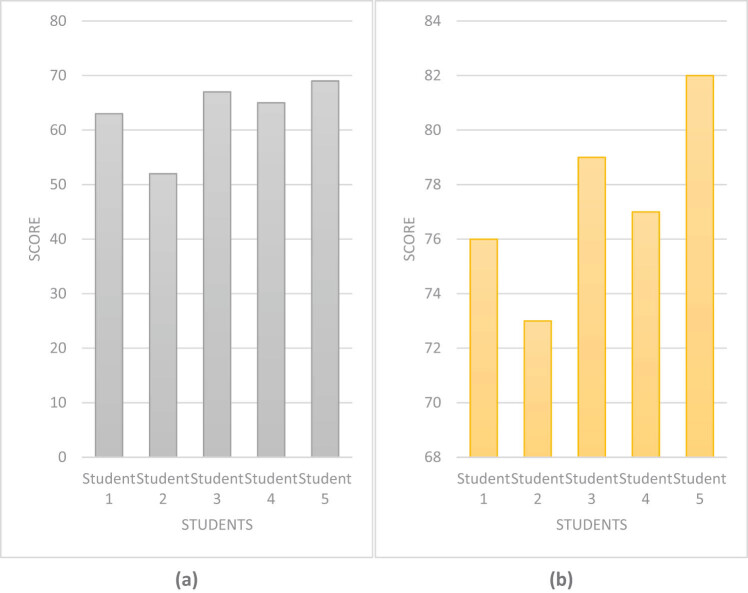
Drug development ability of students using different methods. (a) Drug development ability of students using traditional methods. (b) Drug development ability of students using computerized molecular simulation embedded technology and artificial intelligence.

The most important thing for students to learn about drug research and development is to improve their drug research and development capabilities and encourage them to independently conduct drug research and development. Based on this, this article analyzed students’ drug research and development capabilities. The results are shown in Figure 7.

Figure 7a represents the drug research and development capabilities of students using traditional methods, and Figure 6b represents the drug research and development capabilities of students using computer molecular simulation embedded technology and artificial intelligence. When conducting drug research using traditional drug research methods, Student 5 had the strongest drug research and development ability, with 69 points, while Student 2 had the weakest drug research and development ability, with only 52 points, with a difference of 17 points. However, after using computer molecular simulation embedded technology and artificial intelligence technology, each student’s drug research and development ability has improved, and the difference between the strongest and the weakest was only nine points. This indicates that the gap in drug research and development capabilities among students is gradually decreasing. Overall, students’ drug research and development abilities have been improved by adopting computer molecular simulation embedded technology and artificial intelligence technology.

## 7 Conclusions

To improve the application effect and teaching effect of drug research and development, this article used computer molecular simulation embedded technology and artificial intelligence technology to apply it to the process of drug research and development. At the same time, the experiment was designed to compare and analyze traditional drug research methods and drug research methods using computer molecular simulation embedded technology and artificial intelligence technology. The analysis was conducted from the perspective of the application effect of drug research and the teaching effect of drug research and development, and finally, a feasible conclusion was reached. The use of computer molecular simulation embedded technology and artificial intelligence technology, compared to traditional drug research methods, not only can improve the efficiency and safety of drug research and development but also can improve students’ learning interest and cultivate their drug research and development capabilities. It can be seen that the use of computer molecular simulation embedded technology and artificial intelligence technology can greatly improve the application and teaching effectiveness of drug research.
